# Construction of a New Phage Integration Vector pFIV-Val for Use in Different *Francisella* Species

**DOI:** 10.3389/fcimb.2018.00075

**Published:** 2018-03-14

**Authors:** Hana Tlapák, Kristin Köppen, Kerstin Rydzewski, Roland Grunow, Klaus Heuner

**Affiliations:** ^1^Division 2 (ZBS 2), Cellular Interactions of Bacterial Pathogens, Centre for Biological Threats and Special Pathogens, Robert Koch Institute, Berlin, Germany; ^2^Division 2 (ZBS 2), Highly Pathogenic Microorganisms, Centre for Biological Threats and Special Pathogens, Robert Koch Institute, Berlin, Germany

**Keywords:** *Francisella tularensis*, integrative vector, pFIV-Val, genomic island, episomal, phage

## Abstract

We recently identified and described a putative prophage on the genomic island FhaGI-1 located within the genome of *Francisella hispaniensis* AS02-814 (*F. tularensis* subsp. *novicida*-like 3523). In this study, we constructed two variants of a *Francisella* phage integration vector, called pFIV1-Val and pFIV2-Val (*Francisella* Integration Vector-tRNA^Val^-specific), using the *attL/R-*sites and the site-specific integrase (FN3523_1033) of FhaGI-1, a chloramphenicol resistance cassette and a *sacB* gene for counter selection of transformants against the vector backbone. We inserted the respective sites and genes into vector pUC57-Kana to allow for propagation in *Escherichia coli*. The constructs generated a circular episomal form in *E. coli* which could be used to transform *Francisella spp*. where FIV-Val stably integrated site specifically into the tRNA^Val^ gene of the genome, whereas pUC57-Kana is lost due to counter selection. Functionality of the new vector was demonstrated by the successfully complementation of a *Francisella* mutant strain. The vectors were stable *in vitro* and during host-cell infection without selective pressure. Thus, the vectors can be applied as a further genetic tool in *Francisella* research, expanding the present genetic tools by an integrative element. This new element is suitable to perform long-term experiments with different *Francisella* species.

## Introduction

*Francisella tularensis*, the causative agent of tularemia, is found in a wide range of wild animals and can infect humans, causing various clinical expressions ranging from skin lesions to severe pneumonia, depending on the route of infection (Ellis et al., 2002). Infections in humans are mostly associated with the highly virulent *F. tularensis* subsp. (*Ft*.) *tularensis* and the less virulent subspecies *Ft. holarctica* (*Fth*) (Keim et al., 2007). Opportunistic infections by other *Francisella* species such as *F. hispaniensis* (*Fhis*), *F. novicida* (*Fno*), and *F. philomiragia* (*Fph*) have been reported in individuals with compromised immune systems (Hollis et al., 1989; Clarridge et al., 1996; Whipp et al., 2003). Recently, a new *Francisella* species (*Francisella* sp. strain W12-1067) has been identified in an aquatic habitat in Germany (Rydzewski et al., 2014). Yet it is not clear if the new species will be grouped into the genus *Francisella* or into the new genus “*Allofrancisella*” (Qu et al., 2013, 2016; Challacombe et al., 2017a). So far it is not known if this species is able to infect humans.

We recently identified and described the genomic island (GI) FhaGI-1, located in the genome of *Fhis* AS02-814 (*Ft*. subsp. *novicida-like* 3523) that contains a putative prophage (Schunder et al., 2013). We could show that the GI integrates site specifically into the tRNA^Val^ gene of the genome and that it generates an episomal form in an integrase-dependent manner. Furthermore, we could demonstrate that small variants of FhaGI-1 are able to integrate site specifically into the genome of other *Francisella* species (Rydzewski et al., 2015). Therefore, we decided to create the first *Francisella* phage integration vector on the basis of this GI.

There are a number of tools to manipulate *Francisella* genetically. For the expression of genes and complementation *in trans*, there are several shuttle-vectors derived from the cryptic plasmid pFNL10. Although the second and third generation of these vectors are mostly stable without selective pressure, high copy numbers can still pose a problem (Norqvist et al., 1996; Pomerantsev et al., 2001; Maier et al., 2004; LoVullo et al., 2006). Vectors based on plasmids from *Fph* expand the repertoire of shuttle-vectors and make it possible to use more than one vector per organism (Le Pihive et al., 2009). Chromosomal integration is a way to circumvent the problems associated with high copy numbers. For many bacteria, integration systems based on the site-specific elements of bacteriophages have been described (Lee et al., 1991; Hoang et al., 2000; Lauer et al., 2002). In general, these vectors consist of the site-specific integrase of a bacteriophage together with its *attP-*site (Campbell, 2003), a resistance gene, and a multiple cloning site. For *Francisella* few chromosomal integration systems have been described so far. The existing systems are either based on allelic exchange or a mini-Tn7 vector. Both systems produce transformants that are stable without selective pressure, but they also require helper plasmids or multiple rounds of transformation and selection (Ludu et al., 2008; LoVullo et al., 2009a,b). Phage integration vectors have not been generated for *Francisella* since phages for this organism have not been described before (LoVullo et al., 2009a; Rydzewski et al., 2015). Further cryptic plasmids and a putative conjugative element have been described recently and may be used to generate further plasmids for *Francisella* in the future (Siddaramappa et al., 2014; Challacombe et al., 2017b).

Here we report the construction of two variants of a new phage integration vector pFIV-Val on the basis of the genomic island FhaGI-1 that replicate in *Escherichia coli* and integrate stably and site specifically into the genome of different *Francisella* species.

## Materials and methods

### Strains and growth conditions

Strains used in this study were *E. coli* (DH10B) One Shot® TOP 10 (Invitrogen) and various *Francisella* strains (see Table 1). The *iglC* mutant strain of *Fth* strain LVS was kindly provided by Anders Sjöstedt (Golovliov et al., 2003). For genes and abbriviations used, see Table 1.

**Table 1:** Strains and genetic elements used in this study.

**Strain (abbreviation)**	**Characteristics**	**References**
*Francisella tularensis holarctica* LVS (*Fth* LVS)	Live vaccine strain	ATCC 29684
*Francisella tularensis holarctica* LVS FIV1-Val (*Fth* LVS FIV1-Val)	Strain containing vector FIV1-Val	This work
*Francisella tularensis holarctica* LVS FIV1-Val gfp (*Fth* LVS FIV1-Valgfp)	Strain containing vector FIV1-Val with additional *gfp*-gene	This work
*Francisella tularensis holarctica* LVS FIV2-Val (*Fth* LVS FIV2-Val)	Strain containing vector FIV2-Val	This work
*Francisella tularensis holarctica* LVS ΔiglC (*Fth* LVS ΔiglC)	*iglC* deletion mutant	Golovliov et al., 2003
*Francisella tularensis holarctica* LVS ΔiglC+FIV1-iglC (*Fth* LVS ΔiglC+FIV1-iglC)	*iglC* deletion mutant complemented with FIV1-iglC	This work
*Francisella tularensis holarctica* wild type	Isolated from beaver carcass	Schulze et al., 2016
*Francisella novicida* U112 (*Fno* U112)	Wild type strain	ATCC 15482
*Francisella novicida* U112 FIV1-Val (*Fno* U112 FIV1-Val)	Strain containing vector FIV1-Val	This work
*Francisella novicida* Fx1 (*Fno* Fx1)	Wild type strain	FSC 156
*Francisella* sp. strain W12-1067	Wild type strain	Rydzewski et al., 2014
*Francisella* W12-1067 FIV1-Val	Strain containing vector FIV1-Val	This work
*Francisella hispaniensis* (*Fhis*)	Wild type strain	Whipp et al., 2003
**Gene name/genetic construct**	**Characteristics**	**References**
Integrase (Int)	Site specific integrase of FhaGI-1 (FN3523_1033)	Rydzewski et al., 2015
pGroES	GroES promotor of *Ft. holarctica* LVS	Ericsson et al., 1997
pGroES (W12)	GroES promotor of strain *Francisella* sp. W12-1067	Rydzewski et al., 2014
PRE^*^	*iglA* promotor with PigR response element (PRE)	Ramsey et al., 2015
*attL*-site (tRNA^Val^)	General integration site for FhaGI-1	Rydzewski et al., 2015
*attB* (chromosomal); *attP* (episomal)	Phage attachment sites	Campbell, 2003
*attR*- site	Necessary for the formation of the episomal form of FhaGI-1	Rydzewski et al., 2015
*sacB*	*sacB* gene from *Bacillus subtilis*, coding for a secreted levansucrase	Steinmetz et al., 1985
CmR	Chloramphenicol resistance cassette, *Aeromonas hydrophila*	GenBank accession: AJ973195.1
KmR	Kanamycin resistance cassette	pUC57-Kana (GeneCust)

*E. coli* was cultivated in Luria-Bertani (LB) medium or on LB agar. The antibiotic concentrations used for *E. coli* were chloramphenicol (Cm) 40 μg ml^−1^ and kanamycin (Km) 40 μg ml^−1^. *Francisella* strains were cultivated in medium T (Pavlovich and Mishan'kin, 1987; Becker et al., 2016), on medium T-based agar plates (MT-KH agar: medium T containing 2.4 g l^−1^ of activated charcoal, 14.3 g l^−1^ of agar and 9.5 g l^−1^ of hemoglobin), or on HCA agar (Brain Heart Infusion Agar [Liofilchem, Roseto degli Abruzzi, Italy] with 10% sheep blood). The antibiotic concentrations used for *Francisella* were 10 μg ml^−1^ for chloramphenicol and 12 μg ml^−1^ for kanamycin.

The human macrophage-like cell line U937 (ATCC CRL-1593.2) (growth medium RPMI 1640 + 10% FCS [purchased from PAA, Pasching, Austria]) was used to investigate the intracellular multiplication of *Francisella* strains. U937 cells were cultivated at 37°C and 5% CO_2_.

### Construction of integration vectors

**Figure 1:**
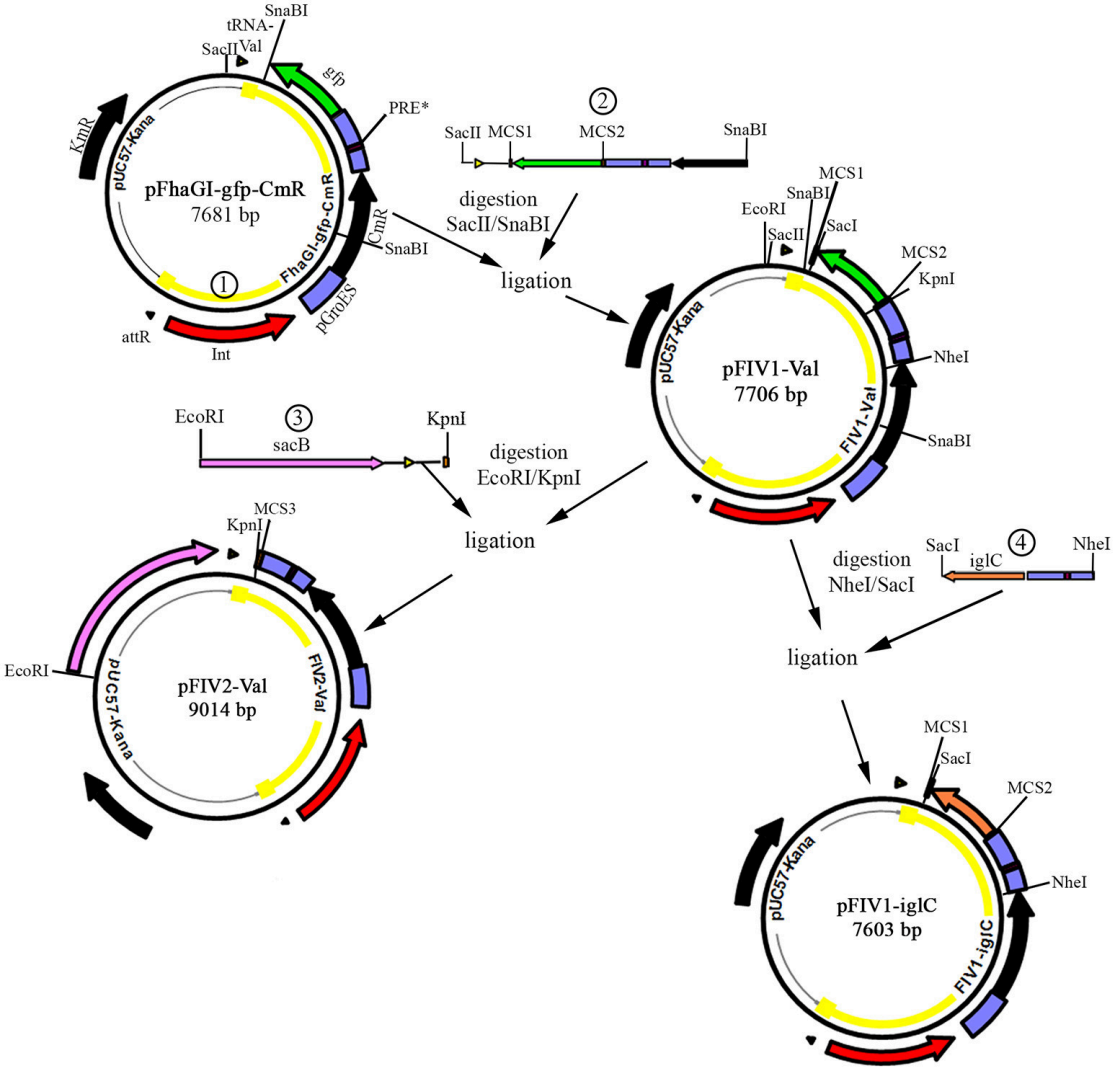
Construction of FhaGI-1-derived vectors and cloning. Vector maps and restriction fragments for the construction of the different vector variants are shown. Antibiotic resistance cassettes for kanamycin (KmR) and chloramphenicol (CmR) are given in black; triangles represent tRNA-Val and *attR* and promotors are shown in blue; the integrase gene is shown in red; genes inserted into the MCS are shown in green (*gfp*) and orange (*iglC*); the *SacB* gene is shown in pink; and the FIV-Val part of the vectors that integrates into the genome of *Francisella* transformants is highlighted by a yellow line. Restriction sites used in this study are indicated. Numbers indicate constructs (1–4) used for cloning, see also Materials and Methods section. Detailed vector maps with primer binding sites are given in Figure S1.

Three different DNA constructs used to generate pFIV-Val vectors were generated by *in vitro* DNA synthesis. DNA synthesis and DNA sequence verification by DNA sequencing were performed by GeneCust (Dudelang, Luxembourg). The different constructs were then used to generate pFIV-Val vectors 1, 2, and pFIV1-*iglC*, using pUC57-Kana as the back-bone (for details, see Table 1 and Figure 1). Construct 1: FhaGI-gfp-CmR is composed of 4,644 bp, exhibiting the tRNA^Val^ gene of FhaGI-1 (Rydzewski et al., 2015), followed by restriction sites for NotI, BclI, and SnaBI, a *gfp* gene with promotor from vector pKK289KmGFP (Bönquist et al., 2008), restriction sites for NotI and SacII, the GroES promotor of *Fth* LVS (pGroES) (Ericsson et al., 1997), the *iglA* promotor with the PigR response element (PRE, underlined bps in PRE^*^), (PRE^*^: AGCTGTATAA ACATTGTGTT ATTGGCGTTA TTAAGGTAAC TT) (Ramsey et al., 2015), the GroES promotor from strain *Francisella* sp. strain W12-1067 (Rydzewski et al., 2014), followed by a Cm resistance cassette (952 bp) with promotor GroES from vector pKK289KmGFP (Bönquist et al., 2008), the integrase of FhaGI-1 (FN3523_1033), and the phage integration site *attR* (47 bp) (Rydzewski et al., 2015).

We introduced the PRE^*^ site into the pFIV-Val vector to introduce a promotor that should be active during intracellular replication of *Francisella*. It has been published that the PRE element is an activator sequence for the expression of virulence genes, including genes (e.g., *iglC*) present on the *Francisella* pathogenicity island (FPI), and that genes of the FPI are induced during intracellular replication of *Francisella* (Ramsey et al., 2015).

Construct 2: The sequence (2389 bp) of construct 2 (see Figure 1) is identical to the sequence of FhaGI-gfp-CmR except for the restriction sequences surrounding the *gfp* gene, designated MCS1 with restriction sites for NotI, BclI, SacI, AatII, and MCS2 with restriction sites for KpnI, EcoRV, NotI, and NcoI. Construct 3 (in pFIV2-Val): SacB-tRNA-MCS3 (2502 bp) is composed of the complete *sacB* gene (2007 bp) of *Bacillus subtilis* (Steinmetz et al., 1985), the tRNA^Val^ gene and a singular MCS3 including restriction sites for BclI, SacI, AatII, KpnI, EcoRV, NotI, and NcoI (Figure 1). In addition, the *iglC* gene of *Ft. holarctica* LVS (construct 4) was cloned into pFIV1-Val using SacI/NheI leading to pFIV1-*iglC* (Figure 1). The maps of pFIV-Val vectors are given in Figure S1.

For the handling of pFIV-Val vectors in in *E. coli* it is necessary to use chloramphenicol and kanamycin simultaneously to select for clones containing the whole vector. Otherwise, the episomal form, which is generated in *E. coli* will be lost, leading to strains containing only the ‘empty’ vector. This is because the episomal form (FIV-Val) is unable to integrate into the genome of *E. coli* or to replicate in *E. coli*. For further details, see Results and Discussion section.

### DNA techniques and PCR analysis

Plasmid DNA for restriction digestion and PCR analysis was prepared using the Invisorb Plasmid Mini Two Kit (Stratec, Berlin, Germany), and preparation of genomic DNA was done with the Blood & Tissue kit (Qiagen, Hilden, Germany). Restriction enzymes were purchased from New England BioLabs and used according to the manufacturer's protocols (Frankfurt a. M., Germany). PCR was carried out using a Thermocycler TRIO-Thermoblock (Biometra, Göttingen, Germany) and the TopTaq DNA polymerase (Qiagen, Hilden, Germany). Analysis of *E. coli* transformants was done with primer pairs Fha-1^P^/Fha-2^**^ (for pFIV1-Val), SacB_R_out/Fha-2^**^ (pFIV2-Val) and Fha-3^*^/Fha-4^P^, for the presence of the complete construct and Fha-1^P^/Fha-4^P^ (pFIV1-Val) and SacB_R_out/Fha-4^P^ (pFIV2-Val), for presence of the “empty” vector. The presence of the episomal form was shown using primer pair Fha-2^**^/Fha-3^*^. Integration of the vectors into the genome of *Francisella* strains was shown using primer combinations Fha-1/Fha-2^**^ and Fha-3^*^ /Fha-4^*^ (for integration into *Fth* LVS and *Fno* U112) and Fha-1^W12^/Fha-2^**^ and Fha-3/Fha-4^W12^ (integration in *Francisella* sp. W12-1067). All mentioned primers are given in Table 2. In general, initial denaturation was performed at 94°C for 3 min and final extension was performed at 72°C for 10 min. The cycling conditions (35 cycles) were 94°C for 30 s, 57°C for 1 min and 72°C for 1 min, and ~ 100 ng of template DNA was used. Oligonucleotides were obtained from Eurofins MWG Operon (Ebersberg, Germany).

**Table 2:** Primers used in this study.

**Primer**	**T_m_ [°C]**	**Sequence 5′3′(bp)**	**References**
Fha-1	61.9	aatcactccaatagccagtactaagga (27)	Rydzewski et al., 2015
Fha-1^W12^	58.9	cttgcttcaatgactgggttttg (23)	This work
Fha-2^**^	60.1	attagcaatgagcttagcttgttgct (26)	This work
SacB_R_out	58.9	ctacgcagacaaacaatcaacgt (23)	This work
Fha-3^*^	59.3	ctgagaattaagccacttatatcagaat (28)	Rydzewski et al., 2015
Fha-4^*^	63.4	gtaaaacccgttggtcaaccttatcag (27)	Rydzewski et al., 2015
Fha-4^W12^	58.9	atccaggaatctttgtaggagct (23)	This work
M13U (Fha-1^P^)	52.8	gtaaaacgacggccagt (17)	O'shaughnessy et al., 2003
M13R (Fha-4^P^)	54.5	ggaaacagctatgaccatg (19)	O'shaughnessy et al., 2003
iglC_U	58.4	actccgatcttactatgcagct (22)	This work
iglC_R	57.3	gcgagaccattcatgtgaga (20)	This work
RT-FIV-CmR-U	60.3	gaaagacggtgagctggtgata (22)	This work
RT-FIV-CmR-R	60.3	gtgtagaaactgccggaaatcg (22)	This work
RT-FIV-CmR-TM	64.6	catcgctctggagtgaataccacga (25)	This work
Ft-fopA-F	57.9	ttgggcaaatctagcaggtca (21)	Schulze et al., 2016
Ft-fopA-R	60.1	atctgtagtcaacacttgcttgaaca (26)	Schulze et al., 2016
Ft-fopA-TM	64.6	FAM- aagaccaccaccaacatcccaagca-BHQ-1 (25)	Schulze et al., 2016

### Transformation of bacteria

Plasmid DNA was introduced into *E. coli* by thermal shock (30 min on ice, 30 s at 42°C, 2 min on ice) (Invitrogen). After transformation *E. coli* were incubated in LB medium for 1 h at 37°C and then plated onto agar containing 40 μg ml^−1^ of chloramphenicol and 40 μg ml^−1^ of kanamycin. Electroporation of *Francisella* strains was performed using a Gene Pulser system (Bio-Rad, Munich, Germany). Electroporation was done at 2.5 kV, 600 Ω and 25 μF. After transformation *Francisella* were incubated in medium T for 4 h at 37°C and then plated onto MT-KH or HCA agar plates containing 10 μg ml^−1^ of chloramphenicol and when appropriate 5% sucrose.

### Testing the stability of vectors

To test the stability of the different vectors in *Francisella* transformants, they were cultured overnight in 3 ml of medium T with Cm (5 μg ml^−1^). The next day 200 μl of the overnight culture were used to inoculate 3 ml of fresh medium T without antibiotics. Bacteria were passaged in this manner every 12 h. After 10 passages the optical density at 600 nm (OD_600_) of the cultures was adjusted to 1, and cultures were diluted and plated on HCA agar with and without chloramphenicol to determine the number of bacteria still containing FIV-Val. Aliquots of the adjusted cultures were used for preparation of genomic DNA and for Western blot analysis. PCR analysis was performed to determine the presence of the integrated as well as the episomal form of FIV-Val.

### SDS-PAGE and immunoblotting

GFP detection was carried out by sodium dodecyl sulfate-polyacrylamide gel electrophoresis (SDS-PAGE) and Western blotting. The SDS-PAGE assay was performed as described previously (Laemmli, 1970). Equal amounts of aliquots of *Francisella* strains from stability testing (20 μl) were boiled for 10 min in Laemmli buffer. A total of 25 μl of the solution was loaded onto a 12% SDS polyacrylamide gel. Western blotting was carried out using a polyclonal anti-GFP antibody (A-11122, Thermo Fisher Scientific, Darmstadt, Germany) diluted in 1% milk–Tris-buffered saline (TBS) (1:1,000). A horseradish peroxidase-conjugated goat anti-rabbit antibody was used as secondary antibody (1:1,000). Visualization was done using ECL Western blotting substrate (Thermo Fisher Scientific) and X-ray film.

### Intracellular replication in U937 cells and fluorescence microscopy

For differentiation into macrophage-like cells, U937 cells were transferred into fresh RPMI medium containing 10% fetal calf serum (10% FCS), and PMA (phorbol-12-myristate-13-acetate, 1 mg/ml in dH_2_O [P-8139; Sigma-Aldrich Chemie, Munich, Germany]) was added at a concentration of 1:20,000. After incubation for 36 h at 37°C and 5% CO_2_, the supernatant was discarded and adherent cells were washed once with 10 ml of 0.2% EDTA in PBS. Cells were mechanically detached from the flask bottom and adjusted to 5 × 10^5^ cells/ml with RPMI + 10% FCS. To each well of a 24-well plate 1 ml of the cell suspension was added and incubated for 2 h at 37°C and 5% CO_2_ for adhesion.

Overnight cultures of *Francisella* strains were diluted in plain RPMI medium, and the infection was done with a multiplicity of infection (MOI) of 10 (time point 0 h) for 2 h at 37°C and 5% CO_2_. Cells were washed three times with RPMI and incubated with 50 μg ml^−1^ of Gentamycin for 1 h to kill extracellular bacteria. Cells were washed again three times with RPMI and covered with 1 ml of RPMI. To determine the CFU at various time points of infection, coincubations of cells and bacteria were lysed by addition of 10 μl of 10% Saponin (S4521, Sigma-Aldrich Chemie) and serial dilutions were plated on HCA agar.

During the infection fluorescent images were obtained every 24 h using an inverse microscope (Carl Zeiss, Jena, Germany).

### Copy number

Copy numbers of FIV-Val were determined by qPCR-analysis. As target for the vector the *CmR* gene was used and as chromosomal reference the single copy gene *fopA*. Primers and hydrolysis probes are given in Table 2.

qPCRs were conducted in a total volume of 25 μl using the ABI 7500 Real Time PCR System and the TaqMan® Environmental Master Mix 2.0 (Applied Biosystems). Primers and hydrolysis probes were used at a final concentration of 0.3 mM and 0.1 mM, respectively. 5 μl of target DNA were added to each reaction. For each vector three decimal dilutions of DNA (0.1; 0.01; 0.001 ng) were pipetted in duplicate. Reactions were initiated with an incubation at 95°C for 10 min followed by 40 cycles of 95°C for 15 s (denaturation) and 60°C for 60 s (annealing and elongation, detection). Copy numbers were calculated using the ΔCt method.

## Results and discussion

### Construction of pFIV-Val

Recently, we demonstrated that the *att*-sites of FhaGI-1 of *Fhis* AS02-814 in combination with the site-specific integrase are sufficient to generate the episomal form FIV-Val of the vector in *E. coli* and that after transformation into *Fth* LVS the element integrates site specifically into the tRNA-Val gene of transformants (Rydzewski et al., 2015). We now utilized this information to develop phage integration vectors to be used in *Francisella* research, with the idea that the constructs would be integrated site specifically and stable into the genome.

We constructed a first variation of the vector, called pFhaGI-gfp-CmR (7,681 bp) (Figure 1 and Figure S1A). The construct was composed of the following elements (see also Table 1): (1) the *attL*-site (tRNA^Val^) which is the general integration site for FhaGI-1; (2) a *gfp* gene flanked by restriction sites that serves as a place holder for the integration of future genes of interest (at this first stage) and as a control for gene expression during intracellular replication, (3) the *Fth* LVS GroES promotor; (4) the PRE^*^ site that is used for the expression of genes during intracellular replication of *Francisella* in host cells; (5) the GroES promotor of strain *Francisella* sp. W12-1067 (pGroES-W12) for expression of genes in this species; (6) a chloramphenicol resistance marker for the selection process after transformation; (7) the site-specific integrase which is necessary for generating the episomal form and the integration into the *attB*-site (tRNA^Val^) of the acceptor strain (*Francisella* strain of interest) and the *attR*-site which is necessary for the formation of the episomal form of pFhaGI-gfp-CmR. For details, see also Materials and Methods. As a backbone, the plasmid pUC57-Kana was used to allow propagation of the construct in *E. coli*. Note, only the FhaGI-gfp-CmR construct (Figure 1 episomal form, marked in yellow) will be integrated site specifically into the genome of the acceptor strain (Rydzewski et al., 2015).

**Figure 2:**
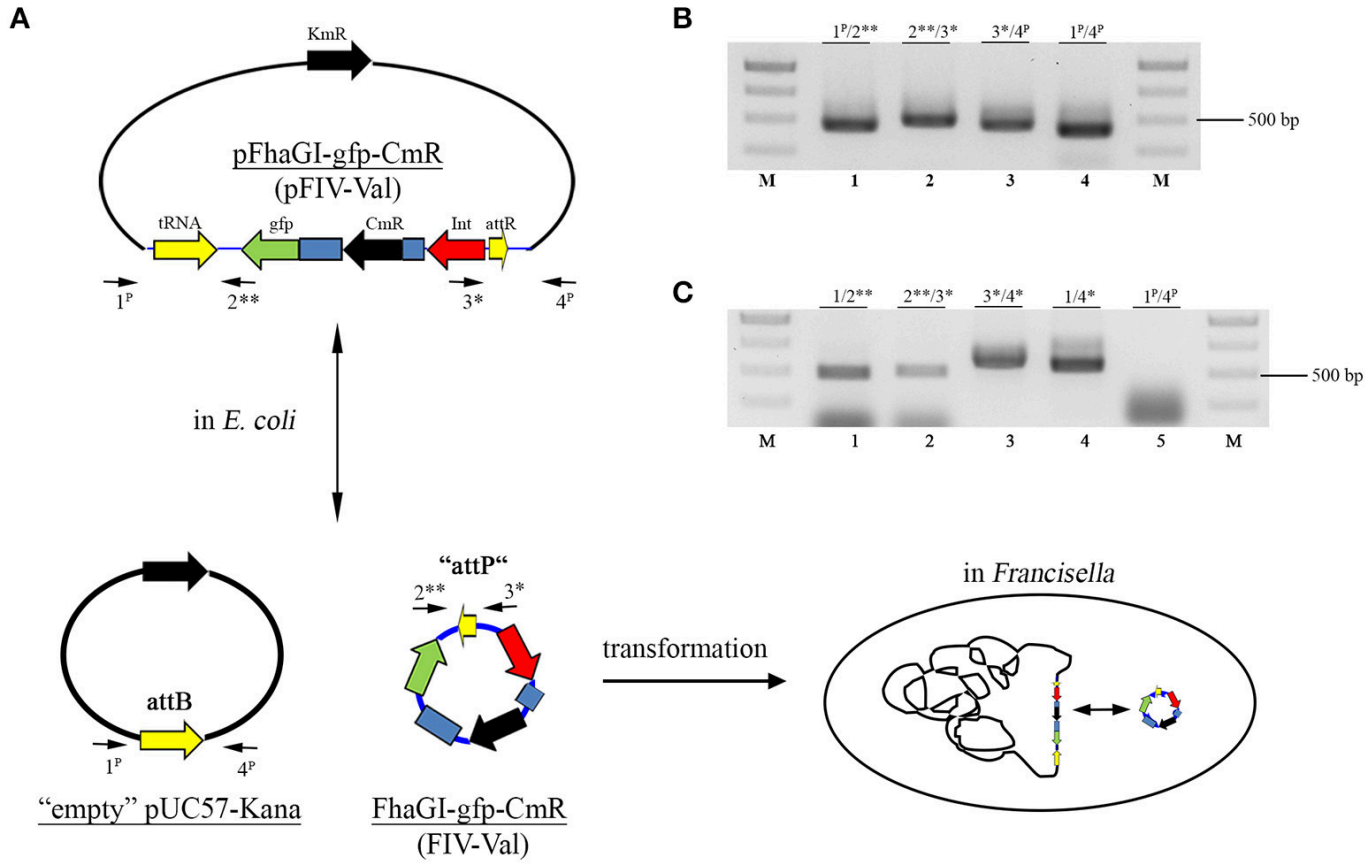
Forms of pFhaGI-1-gfp-CmR. **(A)** In *E. coli* transformants three forms of the vector are present: the complete construct (pFhaGI-gfp-CmR, representing pFIV-Val), the “empty” pUC57-Kana vector with *attB*-site, and the episomal form with *attP*-site (FhaGI-gfp-CmR, representing FIV-Val). After transformation into *Francisella* the FIV-Val part integrates site specifically into the genome. Arrows indicate primers used to detect the different forms of pFhaGI-1-gfp-CmR. **(B)** PCR analysis of an *E. coli* transformant containing pFhaGI-1-gfp-CmR. **(C)** PCR analysis of *Ft. holarctica* LVS transformant containing FhaGI-1-gfp-CmR. Primers Fha-2/3 (Fha-2^**^/Fha-3^*^) show the presence of the episomal form, primers 1/2 and 3/4 (Fha-1/Fha-2^**^ and Fha-3^*^/Fha-4^*^ or Fha-1^P^/Fha-2^**^ and Fha-3^*^/Fha-4^P^) show the chromosomally integrated form of FhaGI-gfp-CmR, whereas primers 1/4 (Fha-1/Fha-4^*^ or Fha-1^P^/Fha-4^P^) show the chromosomal *attB-*site.

The construct was then introduced into *E. coli* by chemical transformation and plated on agar containing chloramphenicol and kanamycin. We isolated plasmid DNA from this strain and analyzed it for the presence of all three forms of pFhaGI-gfp-CmR using primer pairs 1^P^/2^**^ and 3^*^/4^P^ (pFIV-Val), 2^**^/3^*^ (episomal form, FIV-Val), and 1^P^/4^P^ (“empty” vector with *attB*-site) (Figures 2A,B). The PCRs confirmed that all three forms were present which means that the integrase is active in *E. coli*.

The plasmid preparation of pFhaGI-gfp-CmR was introduced into *Fth* LVS by electroporation and plated onto agar plates containing chloramphenicol. One hundred clones were picked and then transferred to plates containing kanamycin for negative selection against transformants still harboring the “empty” vector. Of the picked clones ~30% were kanamycin sensitive and, therefore, maintained only the desired construct (FhaGI-gfp-CmR), the episomal or genomically integrated form of pFhaGI-gfp-CmR (Figure 2A). The loss of the “empty” pUC57-Kana vector was also confirmed by PCR analysis (Figure 2C, lane 5). To test these clones for site-specific integration into the tRNA^Val^ gene they were further analyzed by PCR using species specific (acceptor strain) primers (Figure 2C and Table 2). PCR confirmed that the construct was successfully integrated into the genome of *Fth* LVS (Figure 2C, lanes 1 and 3) and that the episomal form was also present (Figure 2C, lane 2). In addition, the PCR product of about 500 bp using primers 1/4^*^ (Figure 2C, lane 4) confirmed that after excision of FIV-Val, no copy of the GI is left in the genome (see also Rydzewski et al., 2015), indicating that the episomal form is not generated in a replicative way. The results demonstrated that the concept of a phage integration vector works at least in *Fth*.

Subsequently we optimized our vector by introducing an MCS on both sites of the *gfp* gene resulting in vector pFIV1-Val (7,706 bp) (Figure 1 and Figure S1B).

### Functional characterization of pFIV1-Val

**Figure 3:**
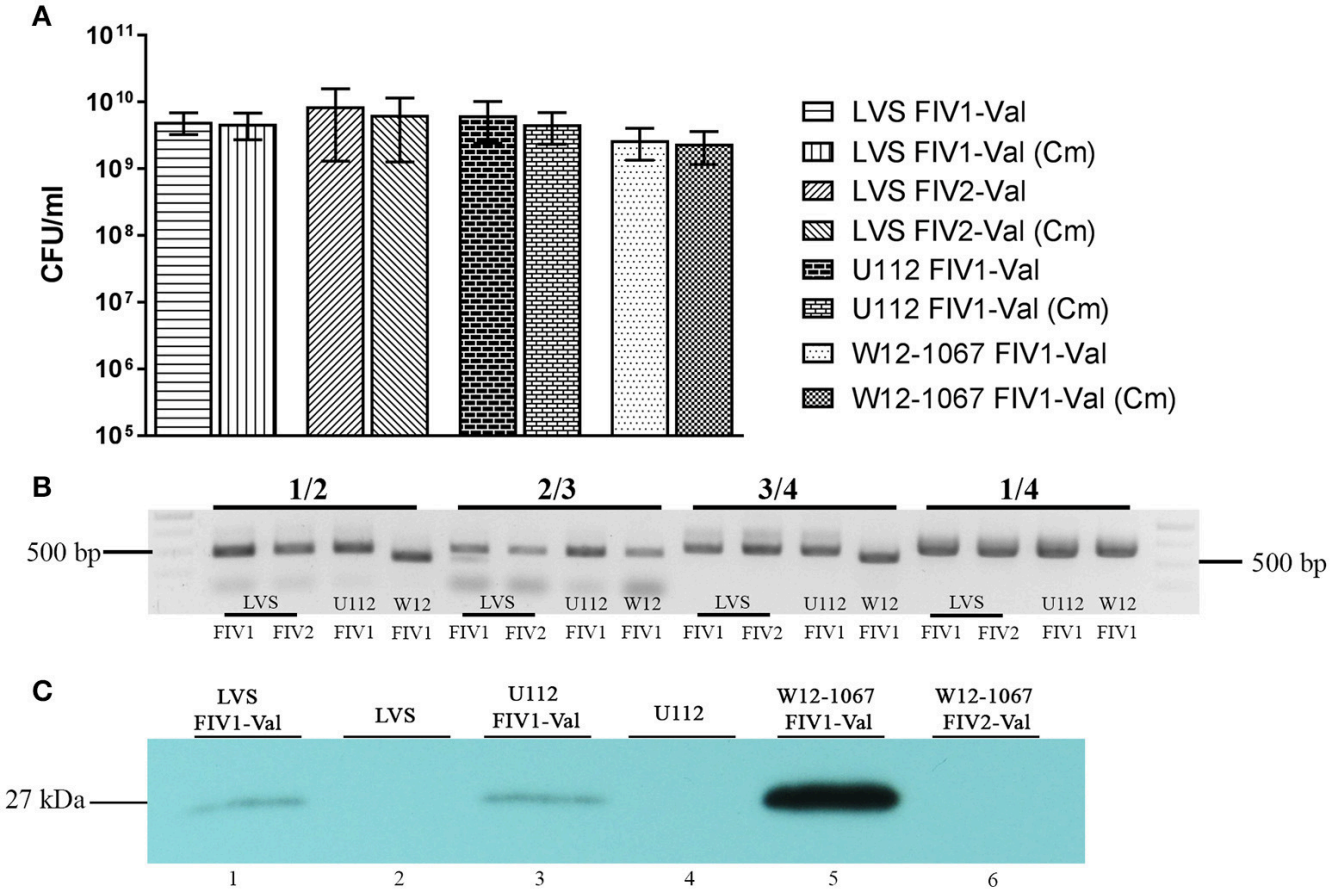
Stability of FhaGI-1-based vectors. Strains *Fth* LVS FIV1-Val, *Fth* LVS FIV2-Val, *Fno* U112 FIV1-Val, and *Francisella* sp. W12-1067 FIV1-Val were passaged 10 times in mediumT without antibiotics. **(A)** CFU of strains on HCA agar and HCA agar containing Chloramphenicol (10 μg ml^−1^) after 10 passages in mediumT without antibiotics; results shown are means with SD of three independent experiments. **(B)** PCR analysis of genomic DNA of strains after 10 passages without antibiotics. Primers 2/3 (Fha-2^**^/Fha-3^*^) show the presence of the episomal form, primers 1/2 and 3/4 (Fha-1 or Fha-1^W12^/Fha-2^**^ and Fha-3^*^/Fha-4^*^ or Fha-4^W12^) show the chromosomally integrated form of FIV-Val, and primers 1/4 (Fha-1/Fha-4^*^ or Fha-1^W12^/Fha-4^W12^) show the chromosomal *attB-*site. **(C)** Western Blot analysis of whole cell lysates with rabbit-α-gfp antibody (1:1,000).

An important characteristic of a vector is its stability without a selective pressure. To test the stability of FIV1-Val in *Fth* LVS without any selective pressure, we cultivated *Fth* LVS harboring FIV1-Val in medium T without antibiotics. After 10 passages, we plated cultures on HCA agar with and without chloramphenicol. As shown in Figure 3A, the CFU of tested strains on agar plates containing chloramphenicol was similar to that on agar without antibiotics, demonstrating that FIV1-Val remained stable without selective pressure. This was further verified by PCR analysis which confirmed the presence of the integrated as well as the episomal form of FIV1-Val (Figure 3B). To test if the gene of interest (e.g., *gfp*) cloned into pFIV1-Val was expressed in the acceptor strain, Western Blot analysis using an anti-GFP antibody was performed. The results demonstrated that the *gfp* gene was expressed (Figure 3C, lane 1), indicating that the used promotor element of FIV-Val is active in *Fth*. The wild type control proves that although the observed band is rather faint it is not due to background binding of the antibody in the strain (Figure 3C, lane 2).

**Figure 4:**
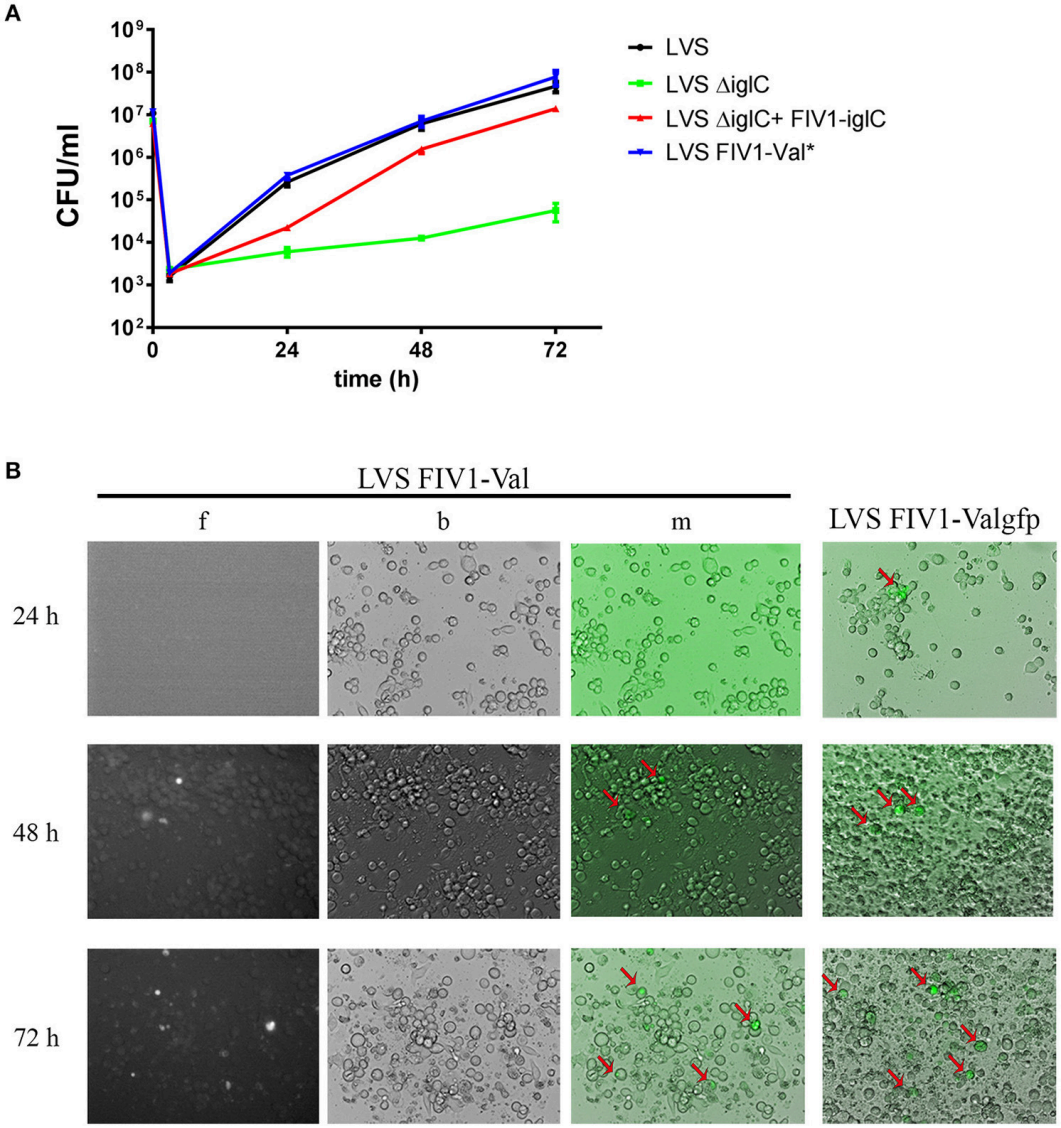
Infection assays using a human macrophage-like cell line (U937). **(A)** Replication of *Fth* LVS wild type (LVS), *Fth* LVS *iglC* mutant (LVS ΔiglC), complemented *Fth* LVS *iglC* mutant (LVS ΔiglC+FIV1-iglC) and *Fth* LVS wild type containing FIV1-Val (LVS FIV1-Val). Cells were infected at an MOI of 10, and CFU was determined by plating on HCA agar every 24 h. Results are mean standard deviations of duplicate samples and are representative of at least 3 independent experiments. ^*^, done only twice. **(B)** Fluorescence microscopy of U937 cells infected with *Fth* LVS FIV1-Val or *Fth* LVS FIV1-Valgfp. f, fluorescence; b, bright field; m, merge.

To demonstrate further that the integration vector is functional in *Francisella*, we used it to complement a specific mutant strain of *Fth*. We chose an *iglC* mutant strain of *Fth* LVS which is known to be unable to replicate within host cells (Golovliov et al., 2003). We cloned the *iglC* gene into pFIV1-Val, resulting in pFIV1-iglC (Figure 1). FIV1-iglC was then successfully integrated into the *iglC* mutant strain, leading to strain *Fth* LVS ΔiglC+FIV1-iglC. The site-specific integration of the construct was confirmed by PCR analysis (data not shown). Then we performed infection assays with this strain as well as the *Fth* LVS wild-type strain and *Fth* LVS FIV1-Val, using the human macrophage-like cell line U937 (Figure 4A). As expected, the *Fth* LVS wild-type strain replicated in the macrophages while the *iglC* mutant strain did not. The complemented *iglC* mutant strain was able to replicate in U937 cells nearly as well as the wild-type strain (Figure 4A). The nearly complete complementation of the *iglC* mutant might be due to the fact that both copies of the *iglC* gene present in the wild-type strain had been inactivated (Golovliov et al., 2003; Lai et al., 2004) and that the expression of the gene from FIV1-Val was not high enough to complement both inactivated genes. However, the intracellular growth defect of the *iglC* mutant strain was complemented. In addition, we could demonstrate that the presence of FIV1-Val alone did not influence the intracellular replication of *Fth* LVS (Figure 4A). The results demonstrated that FIV-Val was stable without selective pressure and could be used to express genes of interest during intracellular replication of *Francisella* in host cells. In addition, since FIV-Val did not influence the ability of *Francisella* to replicate intracellularly in host cells, pFIV-Val can be used for successful complementation of specific mutant strains.

In a further infection assay, we verified whether we could visualize the expression of the *gfp* gene during intracellular replication of *Francisella*. Activity of the *gfp* gene of FIV1-Val during the infection of U937 cells was visualized using a fluorescence microscope. As shown in Figure 4B, the activity of the GFP was low but macrophages containing fluorescent *Fth* LVS FIV1-Val could be demonstrated after 48 h and the number of fluorescent cells increased after 72 h. The results corroborated that the gene of interest cloned into pFIV-Val is expressed during intracellular replication. LoVullo and colleagues observed a similarly low number of fluorescent cells when using a Tn7-based chromosomal integration system to insert a *gfp* gene into the genome of *Fth* LVS (LoVullo et al., 2009a). They attributed the poor visualization to a multitude of factors including promotor strength and improper folding of the GFP. Another factor that might account for the rather weak fluorescent signal could be a low copy number of the gene. Experiments using a strain with an additional *gfp* gene cloned into pFIV1-Val (pFIV-Valgfp) seemed to support the theory of low copy numbers. With this strain a fluorescent signal was visible after 24 h and overall there seemed to be more fluorescent cells (Figure 4B, lane LVS FIV1-Valgfp). Altogether these results further confirmed that FIV1-Val remains stable in *Fth* without selective pressure and that it can be used to manipulate *Fth* strains genetically.

### Functionality test of pFIV-Val in other *Francisella* species

To validate whether pFIV-Val could also be used in other *Francisella* species, we transformed *Fno* U112, *Fno* Fx1, and *Francisella* sp. W12-1067 with pFIV-Val. For all three strains we verified the site-specific integration into the genome and the presence of the episomal form by PCR analysis (Figure 3B). We further analyzed *Fno* U112 and *Francisella* sp. W12-1067 for the stability of FIV1-Val (Figure 3A). In both species the construct remained stable integrated after 10 passages in medium T without antibiotics (Figure 3B, lanes U112 FIV1 and W12 FIV1, respectively). GFP activity in both strains was low but could be confirmed by Western-Blot analysis (Figure 3C, lanes 3 and 5). In *Francisella* sp. W12-1067 the amount of the GFP protein was higher than in both other species investigated, suggesting that the cloned promotor element is highly active in this *Francisella* species. However, the differences in GFP activity could be due to differences in vector copy number, expression or in protein stability (improper folding, LoVullo et al., 2009a). In our hands, similar results were also obtained with plasmids harboring a pGroES-*gfp* gene (unpublished results).

We also successfully introduced pFIV-Val into a wild-type strain of *Fth* (isolated from a beaver, Schulze et al., 2016) (data not shown). These results show that pFIV1-Val is suitable as an integration vector in different species and strains of *Francisella*.

### Further improvements and determination of the copy number of pFIV-Val

To simplify selection of FIV-Val-positive clones after transformation, we decided to employ the idea of a negative selection step and introduced the *sacB* gene of *Bacillus subtilis* into that part of pFIV1-Val which does not integrate into the genome of transformants. The *sacB* gene codes for a secreted levansucrase which is toxic for Gram-negative bacteria when expressed in the presence of sucrose (Steinmetz et al., 1983, 1985). Using the levansucrase, only one selection step of transformants is needed since clones still harboring the “empty” or complete vector will die in the presence of sucrose. The construct “SacB-tRNA-MCS3” was cloned into pFIV1-Val, leading to the second vector called pFIV2-Val (9,014 bp). In addition, to generate a standard cloning vector pFIV2-Val, the *gfp* gene has been deleted and a singular MCS 3 has been introduced instead (Figure 1 and Figure S1D).

After transformation of pFIV2-Val into *Fth* LVS and selection on agar plates containing chloramphenicol and sucrose, only FIV2-Val-positive clones with the integrated form of FIV2-Val could be detected by PCR analysis (Figure 3B and data not shown). This demonstrates that the selection on sucrose was very efficient, thus eliminating the need for a second selection step. FIV2-Val was tested for its stability as described for FIV1-Val. The vector remained stable without selective pressure (Figure 3B, lanes LVS FIV2). However, since the integrase is still located on the FIV2-Val part of the vector, the episomal (excised) form of FIV-Val is still generated and detectable (Figure 3B, primers 2/3). Earlier we could show that the integrase is sufficient for the excision of a small variant of FhaGI-1, but excision still occurred in *Fth* LVS of an element missing the site-specific integrase. This may be due to the presence of further integrases or RecA in the genome sequence of the acceptor strain (Lesic and Carniel, 2005; Rydzewski et al., 2015). We obtained a comparable result for a genomic island (LpcGI-2) of *Legionella pneumophila* in which a similar mechanism was used for the excision of the GI from the genome (Lautner et al., 2013).

First qPCR analyses to quantify the copy number of the FIV-Val constructs (see section Materials and Methods) suggest that the copy number of FIV-Val in *Fth* LVS was 3.6 for both FIV1-Val and FIV2-Val (see Table S1). The results demonstrated that FIV-Val behaves in *Francisella* like a low-copy vector.

## Conclusion

In this study we constructed two variants of a new phage integration vector (pFIV1-Val and pFIV2-Val), derived from FhaGI-1 of *Fhis* AS02-814 for the use in different *Francisella* species. Both constructs integrate site specifically into the genome of the acceptor species at the *attB*-site localized within the tRNA^Val^ gene and remain stable without selective pressure. The introduction of a levansucrase into pFIV2-Val simplified the selection process of FIV-Val-positive strains after transformation of the acceptor with pFIV-Val. qPCR analysis suggests that there are about 3.6 copies of FIV-Val in *Fth* LVS. We used pFIV1-Val to complement successfully an *iglC* mutant strain of *Fth* LVS and could demonstrate that an introduced ‘gene of interest’ (*gfp* gene) was active in three different *Francisella* species.

GFP activity was not high in *Fth*, but the advantage of pFIV-Val is the site-specific integration, the low copy number and the stability without any selective pressure. In contrast to other plasmids or integration systems, pFIV-Val can be used without the help of “helper-plasmids” and in combination with other expression vectors, since no origin of replication is present on FIV-Val. Furthermore, pFIV-Val is usable in different *Francisella* strains of *Fth* and *Fno* and also different *Francisella* species. Thus, our results demonstrate that FhaGI-1-derived vectors can be used as a further genetic tool in *Francisella* research. With this new integration vector we now are able to perform research (in the laboratory) on persistence and reservoir research with *Francisella* spp. in long-term experiments.

## Author contributions

KH designed the study and RG provided facility and equipment. HT, KK, and KR performed the experiments. HT and KH wrote the paper.

### Conflict of interest statement

The authors declare that the research was conducted in the absence of any commercial or financial relationships that could be construed as a potential conflict of interest.

## Supplementary material

Figure S1 Detailed vector maps of FhaGI-derived vectors. Given are vector maps for the different versions of the vector. Antibiotic resistance cassettes for kanamycin (KmR) and chloramphenicol (CmR) are given in green; yellow triangles represent tRNA-Val and *attR* and promotors are shown in blue; the integrase gene is shown in red; the MCS is given in orange and the *SacB* gene is shown in pink; the FIV part of the vectors that integrates into the genome of *Francisella* transformants is highlighted by a yellow line; primer binding sites are indicated by blue arrows; and restriction sites of the MCS and others used in this study are indicated. Primer binding positions are given in the table below each map; restriction enzymes that do not cut the vectors are given in a list below each vector version. **(A)** Vector map of pFhaGI-gfp-CmR, **(B)** vector map of pFIV1-Val, **(C)** restriction digestion of the three pFIV-Val vectors with NheI/SacI. ^*^, desired fragment; °, episomal form; [], empty vector; x, cut-out fragment, and **(D)** vector map of pFIV2-Val.

Table S1 Data of qPCR analysis determining the copy number of FIV-Val.
