# Supplementary material for: Construction of a New Phage Integration Vector pFIV-Val for Use in Different Francisella Species
Source: Front Cell Infect Microbiol. 2018 Mar 14;8:75. doi: 10.3389/fcimb.2018.00075 (PMC5861138; doi:10.3389/fcimb.2018.00075)
Supplement: Figure S1 — Detailed vector maps of FhaGI-derived vectors. Given are vector maps for the different versions of the vector. Antibiotic resistance cassettes for kanamycin (KmR) and chloramphenicol (CmR) are given in green; yellow triangles represent tRNA-Val and attR and promotors are shown in blue; the integrase gene is shown in red; the MCS is given in orange and the SacB gene is shown in pink; the FIV part of the vectors that integrates into the genome of Francisella transformants is highlighted by a yellow line; primer binding sites are indicated by blue arrows; and restriction sites of the MCS and others used in this study are indicated. Primer binding positions are given in the table below each map; restriction enzymes that do not cut the vectors are given in a list below each vector version. (A) Vector map of pFhaGI-gfp-CmR, (B) vector map of pFIV1-Val, (C) restriction digestion of the three pFIV-Val vectors with NheI/SacI. *, desired fragment; °, episomal form; [], empty vector; x, cut-out fragment, and (D) vector map of pFIV2-Val. [file DataSheet1.DOCX]

1. **pFhaGI-gfp-CmR Vector Map**

| **primer name** | **primer sequence (5‘- 3‘** | **primer position** |
| --- | --- | --- |
| M13 U | GTAAAACGACGGCCAGT | 7650- 7665 |
| M13 R | GGAAACAGCTATGACCATG | 4706- 4688 |
| Fha 2** | ATTAGCAATGAGTTAGCTTGTTGCT | 398- 373 |
| Fha 3* | CTGAGAATTAAGCCACTTATATCAGAAT | 4252- 4279 |

Enzymes that do not cut:

_________________________________________________________

AarI, AatII, Acc65I, AfeI, AhdI, AscI, AvrII, BaeI, BaeI, BbvCI, BcgI, BcgI, BglII, BmgBI, BmtI, BplI, BseRI, BsgI, BsiWI, BspMI, BssHII, BstEII, BstXI, Bsu36I, EcoRV, FseI, FspAI, KpnI, MluI, NheI, PmeI, PpuMI, PshAI, PsrI, PsrI, RsrII, SanDI, SexAI, SfiI, SgrAI, SrfI, XcmI, XhoI, ZraI

1. **pFIV1-Val Vector Map**

| **primer name** | **primer sequence (5‘- 3‘** | **primer position** |
| --- | --- | --- |
| M13 U | GTAAAACGACGGCCAGT | 7675- 7691 |
| M13 R | GGAAACAGCTATGACCATG | 4731- 4713 |
| Fha 2** | ATTAGCAATGAGTTAGCTTGTTGCT | 398- 373 |
| Fha 3* | CTGAGAATTAAGCCACTTATATCAGAAT | 4277- 4304 |

Enzymes that do not cut:

_________________________________________________________

AarI, AfeI, AhdI, AscI, AvrII, BaeI, BaeI, BbvCI, BcgI, BcgI, BglII, BmgBI, BplI, BseRI, BsgI, BsiWI, BspMI, BssHII, BstEII, BstXI, Bsu36I, FseI, FspAI, MluI, PmeI, PpuMI, PshAI, PsrI, PsrI, RsrII, SanDI, SexAI, SfiI, SgrAI, SrfI, XcmI, XhoI

1. **Restriction digestion of pFIV-Val vectors**

**
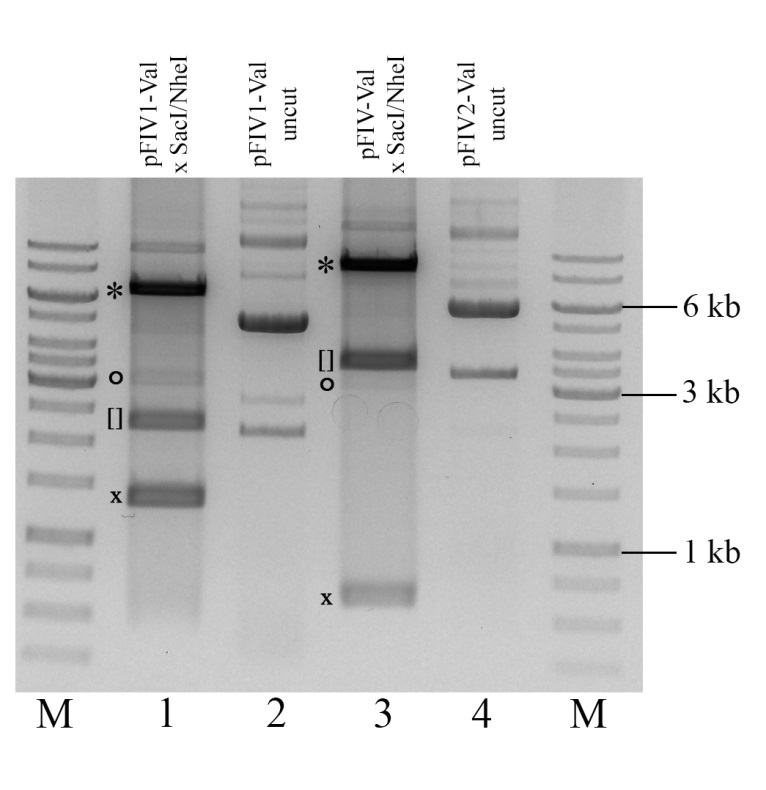
**

Since all three forms of the vector are present in *E. coli*, restriction digestion of pFIV-1 Val with two enzymes (e.g. SacI/NheI) led to more than the two bands expected, in this case the *gfp* gene (~1.4 kb) and the complete construct without *gfp* (~6.4 kb). Since the restriction sites are located on the part of the vector that forms the episomal form, an additional band representing the episomal form (without *gfp*) was present (~3.1 kb). A further band is formed by the 'empty' vector, so that in this case four bands were obtained when cutting pFIV1-Val (see Fig. S1C).

1. **pFIV2-Val Vector Map**

| **primer name** | **primer sequence (5‘- 3‘** | **primer position** |
| --- | --- | --- |
| M13 U | GTAAAACGACGGCCAGT | 6930- 6946 |
| M13 R | GGAAACAGCTATGACCATG | 3986- 3968 |
| Fha 2** | ATTAGCAATGAGTTAGCTTGTTGCT | 398- 373 |
| Fha 3* | CTGAGAATTAAGCCACTTATATCAGAAT | 3532- 3559 |

Enzymes that do not cut:

_________________________________________________________

AarI, AfeI, AhdI, AscI, AvrII, BbsI, BbvCI, BcgI, BcgI, BglII, BmgBI, BsaI, BseRI, BsiWI, BssHII, BstEII, BstXI, Bsu36I, FseI, FspAI, MfeI, MluI, PmeI, PmlI, PpuMI, PshAI, PsrI, PsrI, RsrII, SacII, SanDI, SexAI, SfiI, SgrAI, SrfI, XcmI, XhoI

**Fig. S1: Detailed vector maps of FhaGI-derived vectors.** Given are vector maps for the different versions of the vector. Antibiotic resistance cassettes for kanamycin (KmR) and chloramphenicol (CmR) are given in green; yellow triangles represent tRNA-Val and *attR* and promotors are shown in blue; the integrase gene is shown in red; the MCS is given in orange and the *SacB* gene is shown in pink; the FIV part of the vectors that integrates into the genome of *Francisella* transformants is highlighted by a yellow line; primer binding sites are indicated by blue arrows; and restriction sites of the MCS and others used in this study are indicated. Primer binding positions are given in the table below each map; restriction enzymes that do not cut the vectors are given in a list below each vector version. A) vector map of pFhaGI-gfp-CmR, B) vector map of pFIV1-Val, C) restriction digestion of the two pFIV-Val vectors with NheI/SacI. *, desired fragment; °, episomal form; [], empty vector; x, cut-out fragment, and D) vector map of pFIV2-Val.
